# Resveratrol affects the expression of glutamate cysteine ligase in the kidneys of aged rats

**DOI:** 10.3892/etm.2014.1664

**Published:** 2014-04-03

**Authors:** JIANGSHUI YUAN, ZONGLIANG ZHANG, LI LI, WEIQING SONG

**Affiliations:** 1 Clinical Laboratory, Qingdao Municipal Hospital, Qingdao, Shandong 266011, P.R. China; 2 Department of Urology, Qingdao Municipal Hospital, Qingdao, Shandong 266011, P.R. China

**Keywords:** glutamate cysteine ligase, glutathione, resveratrol, rat, kidney

## Abstract

The aim of the present study was to evaluate the effect that a dietary intake of resveratrol (RSV) had on the expression of glutamate cysteine ligase (GCL) in the kidneys of aged rats. Young, middle-aged and aged rats were each randomly divided into two groups. The control groups were fed a controlled diet and the experimental groups received a controlled diet supplemented with RSV. GCL activity levels in the kidneys were determined. Protein content and relative gene expression levels of the two subunits of GCL were evaluated by western blot analysis and quantitative polymerase chain reaction, respectively. GCL activity levels significantly increased in the kidneys of aged rats fed the RSV-supplemented diet. In addition, RSV markedly increased the protein content and relative mRNA expression levels of the GCL subunits in the kidneys of aged rats. These observations have important implications for the development of therapeutic agents for the kidneys that may enable the elderly population to combat oxidative stress.

## Introduction

Glutathione (GSH) is a tripeptide that functions as an antioxidant. It is a coenzyme that is involved in the detoxification of endogenous and exogenous compounds (1). GSH conjugation with oxidants is catalyzed by the enzyme glutathione *S*-transferase. GSH-dependent detoxifying reactions protect cells from oxidative damage, but consequently reduce intracellular GSH levels. The replenishment of GSH is achieved by recycling and biosynthesis (2), which is regulated by substrate availability and the synthesis rate. Intracellular synthesis of GSH occurs by two consecutive adenosine triphosphate (ATP)-dependent enzymatic reactions. In the first reaction, glutamate is coupled with cysteine to form γ-glutamylcysteine (γ-GC). This is catalyzed by glutamate cysteine ligase (GCL), which is the rate-limiting enzyme of GSH biosynthesis. In the second reaction, γ-GC is coupled with glycine to form GSH and this is catalyzed by GSH synthetase (2).

Aging is a process of chronic oxidative stress and has been shown to affect GSH levels in a tissue-specific manner (3). In type I skeletal muscles, GSH levels increase with aging (4,5). In the soleus muscle under stress, aging impairs the expression of the catalytic subunit of GCL (GCLC) (2). This decreased GCL activity is associated with the reduction of GCLC protein expression (2)

A previous study observed that certain plant extracts affect GCLC content (6). Resveratrol (RSV; 3,5,4′-trihydroxystilbene) is a polyphenolic bioactive substance with multiple functions that occurs naturally in several plant species, including grapevines and berries (7). Accumulating evidence has indicated that RSV has anticarcinogenic, anti-inflammatory, antimicrobial, antiviral and antioxidant properties that may be relevant to chronic diseases and/or longevity in humans (8,9). In addition, RSV has been hypothesized to possess anti-aging activity. For example, RSV has been shown to prolong the lifespan and retard the onset of age-associated markers in a short-lived fish, as well as in invertebrate nematode worms and fruit flies (10–14). Therefore, the aim of the current study was to evaluate the effect that a dietary intake of RSV had on the expression of GCLC and the modifier subunit of GCL (GCLM) in the kidneys of aged rats.

## Materials and methods

### Animals and treatments

All animal experiments were conducted according to the methods of Yuan *et al* (7). The animal experimental procedures were approved by the Ethics Committee of the Laboratory Animal Administration of Shandong Province (permit number, SD2007695; Jinan, China). Specific pathogen-free male Wistar rats (*Rattus norvegicus*), aged 8 weeks (no. 20080002), were purchased from Shandong Lukang Pharmaceutical Co., Ltd. (Jining, China) and housed in an environmentally controlled atmosphere (temperature, 22°C; relative humidity, 56%) with a 12-h light/dark cycle. The rats had free access to water and their respective diets and were provided with shredded wood floor bedding for social activity. Five rats were housed in each cage until the age of 3 months and thereafter two rats were housed per cage. Wistar male rats have a life span of 24–30 months; thus, 12- and 21-month-old rats were used as models of middle-aged and aged rats, respectively.

Young (2 months old; n=20), middle-aged (12 months old; n=20) and aged (21 months old; n=20) rats were each randomly divided into two groups of 10 animals. Rats in the control groups (n=10 per age group) were fed a controlled diet (complete semisynthetic columniformed diet containing 18% crude proteins and 5% cellulose, following the recommendations of the Chinese Association For Laboratory Animal Sciences), while rats in the experimental groups (n=10 per age group) were fed the controlled diet supplemented with RSV (Tianjin Jianfeng Natural Product R&D Co., Ltd., Tianjin, China) at a dose of 22 mg/kg of body weight continuously for 45 days. RSV was dissolved in distilled water to reach a concentration of 10 mg/ml. A volume of RSV solution that correlated with the body weight of each rat was injected into a small piece of the controlled diet. The food injected with RSV was then administered to the rats, ensuring that the rats ate the food completely. Drinking bottles with fresh mineral water were replaced daily. Throughout the 45-day study period, rats were allowed free access to the selected diet and drinking water. At the end of the study, rats were sacrificed under anesthesia and the kidneys were removed under sterile conditions.

### GCL activity assay

GCL activity assays were performed according to the method of Chen *et al* (2). GCL activity levels were determined by a fluorescence assay. Frozen rat kidneys were homogenized in 20 mM Tris, 1 mM EDTA, 250 mM sucrose, 20 mM sodium borate and 2 mM serine (TES/SB buffer). Homogenates were centrifuged at 10,000 × g at 4°C for 10 min. The supernatants were collected and then centrifuged again at 15,000 × g at 4°C for 20 min. Protein concentrations in the supernatants were determined using a bicinchoninic acid (BCA) protein assay kit (Beyotime Institute of Biotechnology, Shanghai, China) with bovine serum albumin as the standard.

In the GCL activity assay, 30 μl homogenate was added to 30 μl GCL reaction cocktail (400 mM Tris, 40 mM ATP, 40 mM L-glutamic acid, 2 mM EDTA, 20 mM sodium borate, 2 mM serine and 40 mM MgCl_2_) and incubated at 37°C for 5 min. After incubation for 5 min, 30 μl cysteine (30 mM; dissolved in TES/SB buffer) was added and the mixture was incubated for 13 min at 37°C. The enzymatic reaction in the mixture was stopped by precipitating the proteins with 200 mM 5-sulfosalicylic acid (SSA). After placing on ice for 20 min, the mixture was centrifuged at 2,000 × g at 4°C for 10 min. Next, 20 μl supernatant containing the γ-GC product was added to a 96-well plate designed for fluorescence detection. For each assay, 20 μl γ-GC standards containing 30 μl GCL reaction cocktail, 30 μl SSA (200 mM), 30 μl H_2_O and 30 μl γ-GC standard solution (0, 20, 40, 60, 80, 100, 120 and 140 μM γ-GC in TES/SB buffer) were added to the same 96-well plate to generate a standard curve. Next, 180 μl 2,3-naphthalenedicarboxyaldehyde (NDA) was added to each well. The plate was incubated in the dark at room temperature for 30 min. Following incubation, the formation of NDA-γ-GC was measured (472 nm excitation/528 nm emission) using a fluorescent plate reader (GENios Plus; Tecan Ltd., Männedorf, Switzerland). The quantity of γ-GC in each sample was calculated using the standard curve. Values were expressed in mM/min/mg of protein.

### Protein content of GCLC and GCLM

The protein levels of GCLC and GCLM in the kidneys were determined by western blot analysis. Kidneys were homogenized in DNase buffer containing 20 mM Tris (pH 6.8), 1 mM CaCl_2_, 5 mM MgCl_2_ and 150 U/ml DNase I (Takara Bio, Inc., Dalian, China). The homogenate was then placed on ice for 40 min. Next, urea buffer containing 6 M urea, 2% sodium dodecyl sulfate (SDS) and 20 mM Tris (pH 6.8) was added to the homogenate, which was then homogenized again. The homogenate was centrifuged at 600 × g at 4°C for 15 min and the supernatant was collected. Protein concentrations in the supernatant were determined using the BCA protein assay kit. Equal amounts (20 μg for GCLC and 50 μg for GCLM, determined by linear responses of the respective antibody) of protein were loaded onto 12% SDS-polyacrylamide gels and separated by electrophoresis using Mini-Vertical Gel Electrophoresis Units (Bio-Rad, Hercules, CA, USA). Proteins resolved on the gels were transferred to polyvinylidene difluoride (PVDF) membranes using Mini Trans-Blot Electrophoretic Transfer Cells (Bio-Rad) at 110 V for 3 h. A kidney sample was used as an internal control and loaded and transferred on each blot. The band intensity of all the samples was normalized against the intensity of the internal control, thus permitting the comparison of samples across multiple blots.

Protein-bound PVDF membranes were incubated overnight at 4°C with polyclonal GCLC antibodies (1:3,000; Abcam, Cambridge, UK) or monoclonal GCLM antibodies (1:3,000; Abcam). Blots probed with GCLC or GCLM antibodies were then incubated with secondary antibodies (goat anti-rabbit IgG; 1:3,000; Beijing Kangwei Technology Group Co., Ltd., Beijing, China) conjugated with peroxidase for 1 h at room temperature. The substrate, 5-bromo-4-chloro-3-indolyl phosphate *p*-toluidine-nitroblue tetrazolium chloride (Beijing Kangwei Technology Group Co., Ltd., Beijing, China), was used for colorimetric visualization of the immunoreactions on the membranes. The immunoblots were imaged using a JS-680D automatic gel imaging analyzer (Shanghai Peiqing Science and Technology Co., Ltd., Shanghai, China). The intensity of the immunoreactions on the blots was quantified using Quantity One software (SensiAnsys; Shanghai Peiqing Science and Technology Co., Ltd.).

### Quantitative polymerase chain reaction (qPCR)

Kidney samples were pulverized using liquid nitrogen. Total RNA was extracted from the samples using TRIzol reagent (Takara Bio, Inc.) and treated with RNase-free DNase (Sangon Biotech, Co., Ltd., Shanghai, China) to remove genomic DNA contamination. Next, 1 μg RNA was reverse transcribed to cDNA using a reverse transcription system kit (Sangon Biotech Co., Ltd.). Gene expression levels of GCLC and GCLM were evaluated by qPCR. Primers for GCLC were as follows: Forward, 5′-CTGAGGCAAGATACCTTTATGACC-3′ and reverse, 5′-GTAGCTATCTATTGAGTCATACCGAGAC-3′. Primers for GCLM were as follows: Forward, 5′-CTGTACCAGTGG GCACAGGTAA-3′ and reverse, 5′-TTGGGTCATTGTGAG TCAGTAGC-3′. The mRNA expression level of β-actin was also detected as an internal control for each sample. For β-actin, the following primers were used: Forward, 5′-ACA TCCGTAAAGACCTCTATGCCAACA-3′ and reverse, 5′-GTGCTAGGAGCCAGGGCAGTAATCT-3′.

qPCR was performed using a SYBR Green I PCR kit (Takara Bio, Inc.), according to the manufacturer’s instructions, in an ABI PRISM 7500 sequence detection system (PerkinElmer, Norwalk, CT, USA). Amplification conditions were as follows: 95°C for 10 sec, followed by 40 cycles of 95°C for 5 sec and 60°C for 41 sec. Each experiment was performed in triplicate. The PCR products were run in an agarose gel and were confined to a single band of the expected size in all cases. Melting-curve analysis was also performed to ensure the specificity of the products. Relative mRNA expression levels of GCLC and GCLM were determined using the comparative (2^−ΔΔCt^) method.

### Statistical analysis

Statistical analysis was performed using the Student’s t-test with SPSS software, version 13.0 (SPSS, Inc., Chicago, IL, USA). Analysis was conducted separately for the young, middle-aged and aged rat studies since they were performed independently. Data are expressed as the mean ± standard error. P<0.05 was considered to indicate a statistically significant difference. All figures were created with GraphPad Prism software, version 5.0 (GraphPad Software, Inc., La Jolla, CA, USA).

## Results

### GCL activity

**Figure 1:**
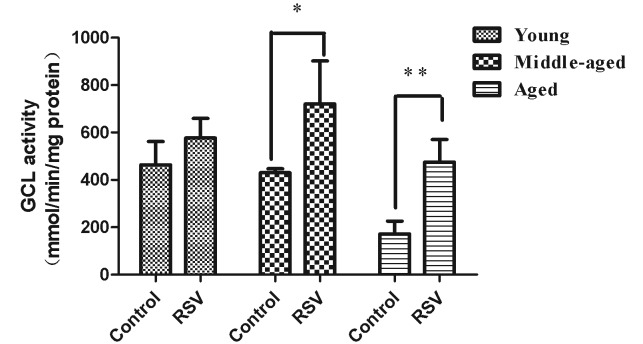
GCL activity levels in the kidneys of young (2 months old), middle-aged (12 months old) and aged (21 months old) rats fed a control diet or a diet supplemented with RSV. GCL activities of the various groups were observed. Values are expressed as the mean ± standard error. ^**^P<0.01 and ^*^P<0.05, RSV vs. control. GCL, glutamate cysteine ligase; RSV, resveratrol.

GCL is the rate-limiting enzyme of GSH synthesis (15). RSV supplementation was not observed to significantly affect the GCL activity levels (Fig. 1) in the kidneys of the young rats (578 ± 83 mmol/min/mg protein) compared with those in the respective control group (425 ± 92 mmol/min/mg protein). As shown in Fig. 1, the GCL activity levels were significantly increased in the kidneys of middle-aged (668 ± 110 mmol/min/mg protein, p=0.026) and aged rats (508 ± 94 mmol/min/mg protein, p=0.001) that were fed the RSV-supplemented diet compared with those in the respective control groups (401 ± 62 mmol/min/mg protein for middle-aged rats and 189 ± 81 mmol/min/mg protein for aged rats).

### Protein content of the GCL subunits

In order to understand the increase of GCL activity levels in the kidneys of aged rats, the protein content of the two subunits of GCL was determined.

**Figure 2:**
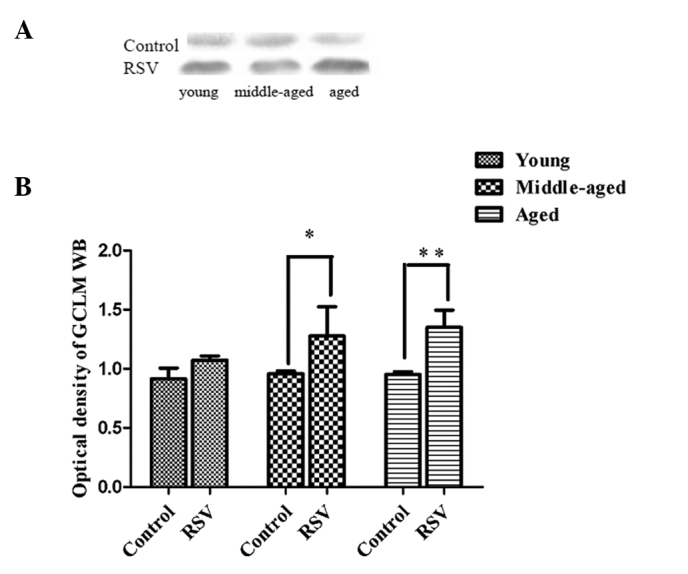
Relative protein content of GCLM. (A) Representative immunoblots and (B) densitometric analysis of GCLM from the kidneys of young (2 months old), middle-aged (12 months old) and aged (21 months old) rats fed a control diet or a diet supplemented with RSV. Values are expressed as mean ± standard error. ^**^P<0.01 and ^*^P<0.05, RSV vs. control. GCL, glutamate cysteine ligase; GCLM, modifier subunit of GCL; RSV, resveratrol; WB, western blot.

GCLM decreases the Km (increases the affinity) for glutamate and ATP and increases the concentration of GSH required for GCL inhibition (Ki) (16). Fig. 2 shows the representative immunoblots and summary of densitometric analysis of GCLM from the kidneys of the rats. In young rats, the relative GCLM content of the kidneys did not change significantly when the RSV-supplemented diet was administered. In middle-aged (1.44 ± 0.83, P=0.016) and aged rats (1.39 ± 0.56, P=0.001) fed the controlled diet supplemented with RSV, the relative GCLM contents in kidneys were greater than the values observed in the respective control groups.

**Figure 3:**
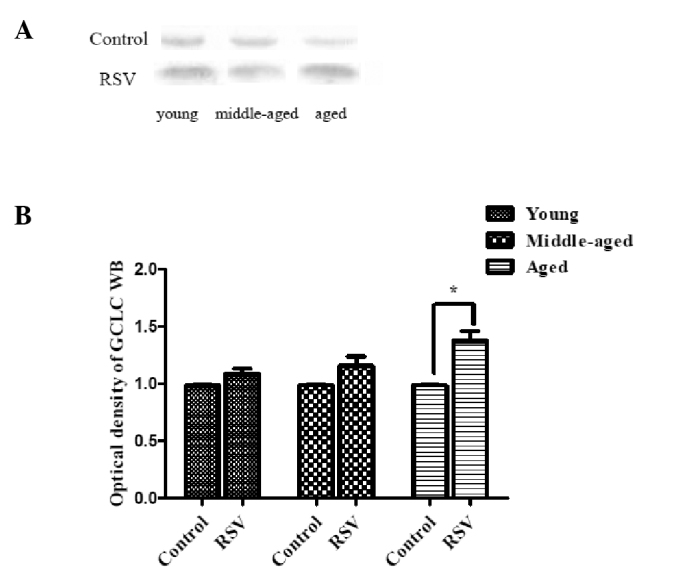
Relative protein content of GCLC. (A) Representative immunoblots and (B) densitometric analysis of GCLC from the kidneys of young (2 months old), middle-aged (12 months old) and aged (21 months old) rats fed a control diet or a diet supplemented with RSV. Values are expressed as mean ± standard error. ^**^P<0.01 and ^*^P<0.05, RSV vs. control. GCL, glutamate cysteine ligase; GCLC, catalytic subunit of GCL; RSV, resveratrol; WB, western blot.

GCLC produces the catalytic function of GCL (16). Fig. 3 shows the representative immunoblots and densitometric analysis of GCLC from the kidneys of the rats. As shown in Fig. 3, the GCLC content of the kidneys was significantly increased in aged rats (1.37 ± 0.38, P=0.012) fed RSV-supplemented diet compared with those in the respective control group. However, no significant change was observed in the young and middle-aged rats.

These results demonstrate the age-associated differences in the protein levels of the GCL subunits in the kidneys from aged rats fed an RSV-supplemented diet.

### Quantification of the mRNA expression levels of the GCL subunits

**Figure 4:**
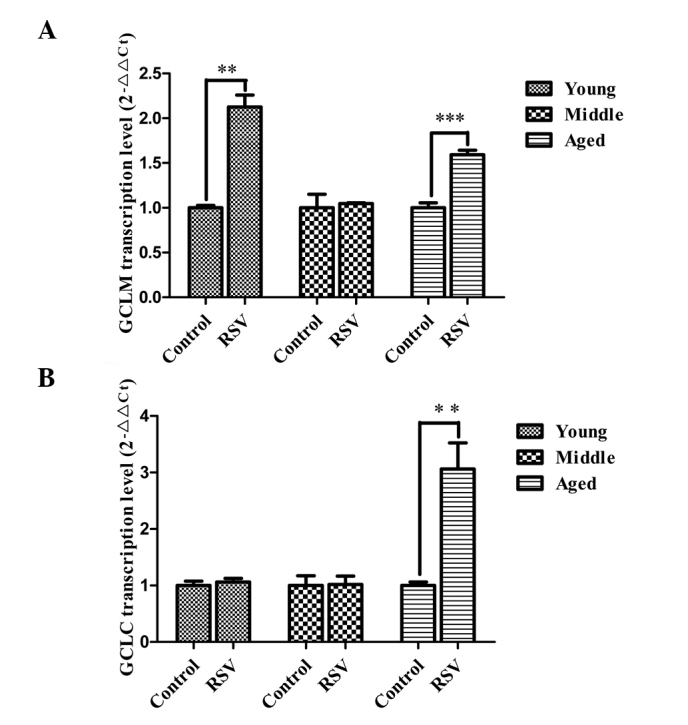
mRNA expression levels of (A) the modifier subunit and (B) the catalytic subunit of glutamate cysteine ligase in kidneys of young (2 months old), middle-aged (12 months old) and aged (21 months old) rats fed a control diet or a diet supplemented with RSV. Quantitative PCR was performed to quantify the expression levels of the GCLM and GCLC genes. Values are expressed as mean ± standard error. ^**^P<0.01 and ^*^P<0.05, RSV vs. control. GCL, glutamate cysteine ligase; GCLC, catalytic subunit of GCL; GCLM, modifier subunit of GCL; RSV, resveratrol; PCR, polymerase chain reaction.

The mRNA expression levels of the GCL subunits were examined in the kidneys of rats fed the control diet or a diet supplemented RSV using qPCR. The results showed that the relative expression levels of GCLM mRNA (Fig. 4A) in kidneys of aged rats (1.55 ± 0.15, P=0.001) fed a diet supplemented with RSV were significantly higher than those in the respective controls; they were also markedly increased in young rats (2.13 ± 0.23, P=0.001) fed the RSV-supplemented diet. A similar effect was not observed in the middle-age rats. As shown in Fig. 4B, the levels of GCLC mRNA expression were significantly increased in aged rats fed an RSV-supplemented diet (2.86 ± 0.31, P=0.001) compared with those in the control; however, a similar change was not observed in the young and middle-aged rats. These results indicate that dietary RSV supplementation is able to induce a significant increase in the mRNA expression levels of GCLC and GCLM in aged rats.

## Discussion

Alterations in GSH metabolism with aging (a condition of chronic oxidative stress) are tissue specific (4,17–23). With aging, organisms exhibit a reduction in the ability to adapt to stress (24–26). Identifying the age-associated changes of GSH homeostasis is important since GSH regulates the redox balance of the cells (2). GCL is the rate-limiting enzyme of GSH synthesis. While the brain and liver are the most studied tissues, few studies have investigated the age-associated changes of GCL in the kidneys. Accumulating evidence indicates that RSV has anticarcinogenic, antiinflammatory, antimicrobial, antiviral and antioxidant properties that may be relevant to chronic diseases and/or longevity in humans (8,9). Thus, the aim of the present study was to determine the mechanism behind the age-associated differences in kidney GCL levels of rats fed a diet supplemented with RSV.

Mechanisms underlying the age-associated upregulation of GSH and GSH-dependent detoxifying enzymes are unknown. However, upregulation is likely to be a compensatory adaptation responding to chronic oxidative stress developed during the aging process (24,27). In the control group in the current study, the GCL activity in the kidneys of the aged rats was observed to be lower compared with the values in the middle-aged and young rats. Although the antioxidant system shows a compensatory adaptation with aging, the ability of aged organisms to positively respond to an additional stress appears to be compromised. The results of the present study indicate that the reduction in GCL activity contributes to a reduction in GSH levels. GCL activity in the kidneys of aged rats fed the RSV diet significantly increased compared with that in the aged rats fed the control diet, and this increase was closely associated with augmentation of the mRNA expression and protein levels of GCLC and GCLM. GCL activity is regulated by the two subunits, GCLC and GCLM. GCLC (73 kDa) contains the active site for the ATP-dependent bond formation between glutamate and cysteine and possesses the catalytic activity of GCL (28). Studies have shown that GCLC alone is necessary and sufficient for γ-GC formation (29,30). GCLM (31 kDa), although having no catalytic activity, enhances enzyme activity by increasing the affinity of GCLC to glutamate and ATP and increasing the concentration of GSH required to inhibit GCL activity. Yang *et al* (30) demonstrated that GCLM homozygous knockout mice had lower GSH levels, increased Km values of GCLC to glutamate and were more sensitive to oxidative stress than their wild-type littermates were. These results demonstrate the important contribution of GCLM in the regulation of GSH levels (2).

In order to understand the increase in GCL activity levels in the kidneys of aged rats fed an RSV diet, the protein levels of the two subunits of GCL were investigated. In the aged rats, it was observed that the GCLC and GCLM protein levels changed significantly. Studies where the GCLC content has been maintained have shown that the addition of GCLM protein increases GCL activity, and conversely, a reduction in GCLM content decreases GCL activity (23,30,31). Previous studies have also indicated that the effect of GCLM on the changes to GCL activity with stress is likely to be dependent on the changes of GCLC content (2).

Increased GCL activity in the kidneys of aged rats fed a diet supplemented with RSV is associated with an increase in GCLC protein and mRNA expression. These results indicate that dietary RSV supplementation is able to modulate GCL activity.

Therefore, the results of the present study highlight the antioxidant properties of RSV mediated via the modulation of GCL activity in the kidneys of aged rats. RSV is a good candidate for further study of antioxidative activity. These observations have important implications in the development of therapeutic agents for the kidneys that may enable the elderly population to combat oxidant stress.

## References

[1] Brown-Borg HM, Rakoczy SG (2005). Glutathione metabolism in long-living Ames dwarf mice. Exp Gerontol.

[2] Chen CN, Brown-Borg HM, Rakoczy SG, Ferrington DA, Thompson LV (2010). Aging impairs the expression of the catalytic subunit of glutamate cysteine ligase in soleus muscle under stress. J Gerontol A Biol Sci Med Sci.

[3] Maher P (2005). The effects of stress and aging on glutathione metabolism. Ageing Res Rev.

[4] Leeuwenburgh C, Fiebig R, Chandwaney R, Ji LL (1994). Aging and exercise training in skeletal muscle: responses of glutathione and antioxidant enzyme systems. Am J Physiol.

[5] Chen CN, Brown-Borg HM, Rakoczy SG, Thompson LV (2008). Muscle disuse: adaptation of antioxidant systems is age dependent. J Gerontol A Biol Sci Med Sci.

[6] Liu XP, Goldring CE, Wang HY (2008). Extract of *Ginkgo biloba* induces glutamate cysteine ligase catalytic subunit (GCLC). Phytother Res.

[7] Yuan J, Lu L, Zhang Z, Zhang S (2012). Dietary intake of resveratrol enhances the adaptive immunity of aged rats. Rejuvenation Res.

[8] Pervaiz S, Holme AL (2009). Resveratrol: its biologic targets and functional activity. Antioxid Redox Signal.

[9] Vang O, Ahmad N, Baile CA (2011). What is new for an old molecule? Systematic review and recommendations on the use of resveratrol. PLoS One.

[10] Valenzano DR, Terzibasi E, Genade T, Cattaneo A, Domenici L, Cellerino A (2006). Resveratrol prolongs lifespan and retards the onset of age-related markers in a short-lived vertebrate. Curr Biol.

[11] Evason K, Huang C, Yamben I, Covey DF, Kornfeld K (2005). Anticonvulsant medications extend worm life-span. Science.

[12] Howitz KT, Bitterman KJ, Cohen HY (2003). Small molecule activators of sirtuins extend *Saccharomyces cerevisiae* lifespan. Nature.

[13] Kang HL, Benzer S, Min KT (2002). Life extension in *Drosophila* by feeding a drug. Proc Natl Acad Sci USA.

[14] Wood JG, Rogina B, Lavu S, Howitz K, Helfand SL, Tatar M, Sinclair D (2004). Sirtuin activators mimic caloric restriction and delay ageing in metazoans. Nature.

[15] Lu SC (2009). Regulation of glutathione synthesis. Mol Aspects Med.

[16] Franklin CC, Backos DS, Mohar I, White CC, Forman HJ, Kavanagh TJ (2009). Structure, function, and post-translational regulation of the catalytic and modifier subunits of glutamate cysteine ligase. Mol Aspects Med.

[17] Hollander J, Bejma J, Ookawara T, Ohno H, Ji LL (2000). Superoxide dismutase gene expression in skeletal muscle: fiber-specific effect of age. Mech Ageing Dev.

[18] Ji LL, Dillon D, Wu E (1990). Alteration of antioxidant enzymes with aging in rat skeletal muscle and liver. Am J Physiol.

[19] Kim HG, Hong SM, Kim SJ (2003). Age-related changes in the activity of antioxidant and redox enzymes in rats. Mol Cells.

[20] Liu R, Choi J (2000). Age-associated decline in gamma-glutamylcysteine synthetase gene expression in rats. Free Radic Biol Med.

[21] Mosoni L, Breuillé D, Buffière C, Obled C, Mirand PP (2004). Age-related changes in glutathione availability and skeletal muscle carbonyl content in healthy rats. Exp Gerontol.

[22] Ohrloff C, Hockwin O, Olson R, Dickman S (1984). Glutathione peroxidase, glutathione reductase and superoxide dismutase in the aging lens. Curr Eye Res.

[23] Zhu Y, Carvey PM, Ling Z (2006). Age-related changes in glutathione and glutathione-related enzymes in rat brain. Brain Res.

[24] Lambertucci RH, Levada-Pires AC, Rossoni LV, Curi R, Pithon-Curi TC (2007). Effects of aerobic exercise training on antioxidant enzyme activities and mRNA levels in soleus muscle from young and aged rats. Mech Ageing Dev.

[25] Siu PM, Pistilli EE, Murlasits Z, Alway SE (2006). Hindlimb unloading increases muscle content of cytosolic but not nuclear Id2 and p53 proteins in young adult and aged rats. J Appl Physiol (1985).

[26] Kayani AC, Morton JP, McArdle A (2008). The exercise-induced stress response in skeletal muscle: failure during aging. Appl Physiol Nutr Metab.

[27] Franco AA, Odom RS, Rando TA (1999). Regulation of antioxidant enzyme gene expression in response to oxidative stress and during differentiation of mouse skeletal muscle. Free Radic Biol Med.

[28] Botta D, White CC, Vliet-Gregg P (2008). Modulating GSH synthesis using glutamate cysteine ligase transgenic and gene-targeted mice. Drug Metab Rev.

[29] Dalton TP, Dieter MZ, Yang Y, Shertzer HG, Nebert DW (2000). Knockout of the mouse glutamate cysteine ligase catalytic subunit (Gclc) gene: embryonic lethal when homozygous, and proposed model for moderate glutathione deficiency when heterozygous. Biochem Biophys Res Commun.

[30] Yang Y, Dieter MZ, Chen Y, Shertzer HG, Nebert DW, Dalton TP (2002). Initial characterization of the glutamate-cysteine ligase modifier subunit Gclm(−/−) knockout mouse. Novel model system for a severely compromised oxidative stress response. J Biol Chem.

[31] Lee JI, Kang J, Stipanuk MH (2006). Differential regulation of glutamate-cysteine ligase subunit expression and increased holoenzyme formation in response to cysteine deprivation. Biochem J.

